# Isolated osseous Rosai-Dorfman disease: a case report and review of literature

**DOI:** 10.1007/s00256-025-04986-3

**Published:** 2025-07-28

**Authors:** Justin J. Sun, Benjamin Emert, Sarah Dry, Fatemeh Abdollahi Mofakham

**Affiliations:** 1 Department of Radiological Sciences, University of California Los Angeles, 757 Westwood Blvd, Suite 1638, Los Angeles, CA USA; 2 Department of Pathology & Laboratory Medicine, University of California Los Angeles, 757 Westwood Blvd, CHS 50-060, Los Angeles, CA USA

**Keywords:** Rosai-Dorfman disease, Histiocytosis, Osseous Rosai-Dorfman disease, Sinus histiocytosis with massive lymphadenopathy

## Abstract

Rosai-Dorfman disease (RDD), also known as sinus histiocytosis with massive lymphadenopathy, is a rare histiocytic disorder characterized by proliferation of non-Langerhans cell phagocytic histiocytes. Most patients present with painless massive cervical lymphadenopathy with associated night sweats, malaise, and fever. Osseous manifestations of RDD are uncommon and primary RDD of the bone without lymphadenopathy is thought to be even rarer. It is important to maintain this entity on a differential as it can mimic an aggressive entity. This is exemplified in our case report of a 24-year-old female patient with an incidental finding of a nonspecific mass in the right hemipelvis which was determined to be isolated osseous Rosai-Dorfman disease after a multidisciplinary investigation.

## Introduction

Rosai-Dorfman disease (RDD), also known as sinus histiocytosis with massive lymphadenopathy, is a rare benign idiopathic proliferative disease characterized by proliferation of non-Langerhans cell phagocytic histiocytes. The etiology of RDD is unknown but speculated to be immune dysregulation in response to an infectious agent such as Epstein-Barr virus (EBV) or associated with IgG4 disease [1, 2].

This entity may present with nodal (lymphadenopathy) or extra-nodal (non-lymph node) manifestations. Nodal involvement most frequently presents as lymphadenopathy within the cervical ganglion chains but lymphadenopathy in other stations can occur [1]. Generalized symptoms such as fevers, chills, weight loss, night sweats, and malaise may be associated. Extranodal involvement occurs in up to 20–40% of cases and may occur with or without the presence of lymphadenopathy [1, 3]. The skin, orbits, nasal cavity, paranasal sinuses, and central nervous system are the most frequently affected extranodal sites with clinical symptoms closely related to the organ system involved.

Osseous involvement of Rosai Dorfman disease occurs in approximately 5–10% of cases [2, 4]. Primary RDD of the bone without lymphadenopathy is thought to be even rarer, occurring approximately 2–8% of all RDD cases, with at least 22 cases having been reported in the literature [2, 5, 6]. These patients often clinically present with swelling and bone or joint pain. Primary osseous RDD has been documented in the axial and appendicular skeleton including the skull, clavicle, femur, tibia, sacrum, and small bones of the hands and feet [7]. Osseous RDD often presents in a nonspecific manner on imaging, typically an intramedullary lytic lesion sometimes with surrounding sclerosis on radiographs and computed tomography (CT). Both non-aggressive and aggressive features such as cortical destruction, periosteal reaction, and sclerosis have been described [8, 9]. The sclerosis has been though to reflect healing [10, 11]. On magnetic resonance imaging (MRI), an infiltrative, bone marrow-replacing process sometimes with cortical thickening and focal breakthrough is often observed. Extra-osseous soft tissue masses are present in up to 71% of cases with non-specific signal intensity, enhancement, and peri-lesional edema characteristics [6]. Its nonspecific imaging features require a broad radiological differential diagnosis including benign and aggressive entities such as Langerhans cell histiocytosis (LCH), lymphoma, osteomyelitis, and primary bone sarcoma, making imaging diagnosis challenging. In this article, we present a case of osseous Rosai-Dorfman disease to emphasize the significance of maintaining a wide differential diagnosis and the crucial role of a multidisciplinary team in ensuring adequate tissue sampling, thorough pathological review, and the use of immunohistochemistry staining to achieve an accurate diagnosis and facilitate appropriate management.

## Methods

A chart review of the presented patient was performed including comprehensive review of radiographic imaging and histology from the bone was retrieved.

## Case report

We present the case of a 24-year-old female patient who endorsed a 1-year history of intermittent right hip pain without improvement despite adherence with physical therapy. She denied any fevers, chills, malaise, or other constitutional symptoms. Physical examination was positive for mild tenderness without a palpable mass, swelling, or erythema. A mass within the right anterior hemipelvis was discovered incidentally on an outside CT obtained for a MVA.

**Fig. 1:**
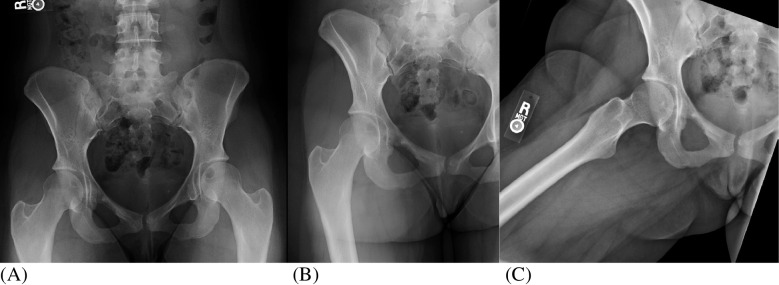
Hip and pelvis radiographs. **A** AP pelvis and **B**, **C** AP and abducted lateral views of the right hip radiographs demonstrate mild bilateral hip dysplasia without associated arthritis. There is a subtle lytic lesion in the right superior pubic ramus as well as an incidental incompletely assessed lucency within the left femoral head, which corresponds to the abnormality on subsequent MR images

**Fig. 2:**
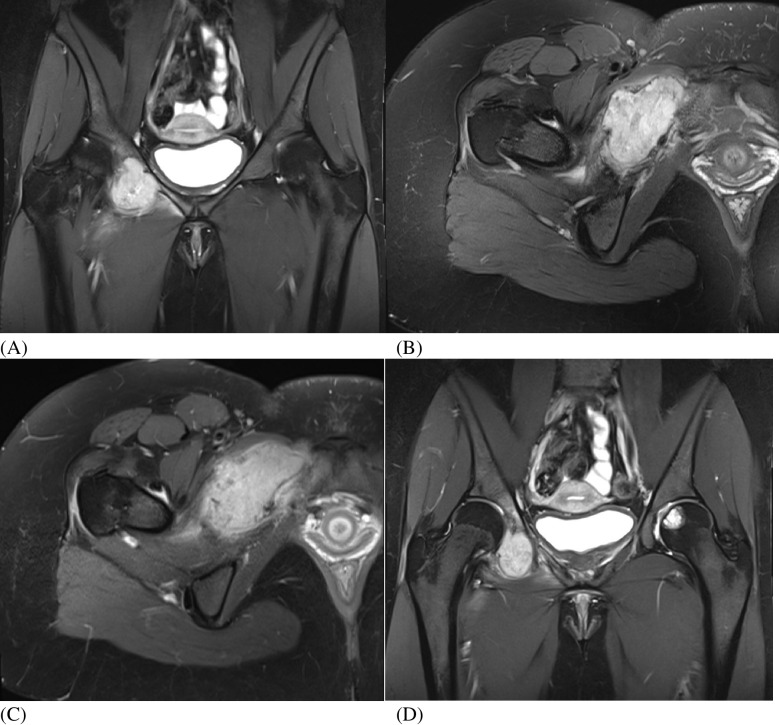
Right hip MRI (Siemens Healthineers MAGNETOM Skyra 3 T). **A**, **B** Coronal T2 FS Dixon_W (TR 3030 ms, TE 66 ms) and axial T2 FS (TR 3460 ms, TE 64 ms) images demonstrate a destructive mildly T2 hyperintense mass in the right superior pubic ramus and acetabulum with a large soft tissue component extending into the adjacent adductor muscles and hip joint. There is cortical breakthrough, and surrounding soft tissue, bone marrow, and periosteal edema. **C** Axial T1 FS Post (TR 804 ms, TE 24 ms, 7.33 mL Gadavist) images show a lobulated soft tissue mass, which demonstrates intense enhancement on T1 post-contrast imaging. **D** Coronal T2 FS Dixon_W (TR 303 ms, TE 66 ms) limited large field-of-view image reveals an additional T2 hyperintense lesion in the left femoral head epiphysis without significant surrounding bone marrow edema and intermediate signal peripheral rind (arrow)

Routine hip and pelvis radiographs including AP view of the pelvis and AP and abducted lateral views of the right hip were initially interpreted as mild hip dysplasia but otherwise unremarkable (Fig. 1). A subtle lucent lesion can be seen in the right superior pubic ramus as well as an incompletely imaged incidental lucent lesion within the left femoral head. No periosteal reaction, identifiable matrix, or cortical destruction was observed. A routine MRI of bony pelvis and right hip was performed on a Siemens Healthineers MAGNETOM Skyra 3 T machine which included the following sequences (Fig. 2): coronal T2 fat saturation (FS) sequences, sagittal proton density (FS), coronal and axial T1 FS post-contrast enhanced, and axial T2 FS. On MRI exam, a destructive mildly T2 hyperintense enhancing mass was identified measuring 31 × 59 × 43 mm and involving the right superior pubic ramus and acetabulum. A large soft tissue component extended into the adjacent adductor muscles and hip joint/acetabular fossa. There was no significant fat signal component demonstrated by a lack of signal drop out on opposed phase and low signal on the Dixon fat sequences. There is prominent surrounding soft tissue, bone marrow, and periosteal edema. An incompletely evaluated T2 hyperintense 23-mm lesion in the left femoral head epiphysis without significant surrounding edema was only partially visualized on this exam. Imaging characteristics were concerning for malignancy and a differential including Ewing’s sarcoma, chondrosarcoma, and Langerhans cell histiocytosis was suggested. Metastatic disease was lower on the differential due to the patient’s age and lack of primary malignancy.

**Fig. 3:**
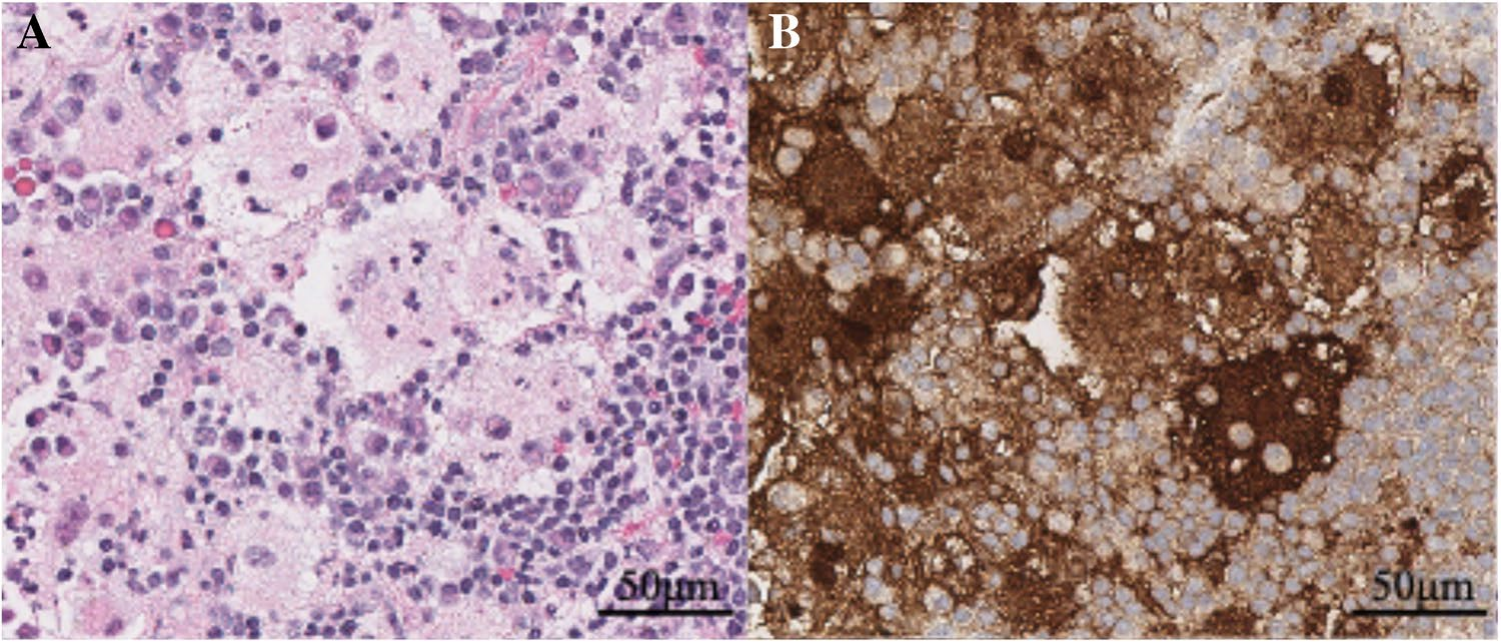
Histopathology of right acetabular biopsy. **A** Hematoxylin and eosin–stained section shows a cluster of lesional histiocytes with round to oval nuclei, prominent nucleoli, abundant eosinophilic cytoplasm, and engulfed inflammatory cells (emperipolesis) in a background of lymphocytes and plasma cells. **B** S100 immunohistochemistry shows positive staining in lesional histiocytes. Scale bars represent 50 μm. Original magnification × 400 for (**A**) and (**B**)

An uncomplicated ultrasound-guided core biopsy of the right acetabular mass was performed. Histopathologic examination revealed clusters of large histocytes with vesicular chromatin and abundant clear to eosinophilic cytoplasm. These histiocytes demonstrated the characteristic finding of emperipolesis, with intact leukocytes engulfed within their cytoplasm (Fig. 3A). By immunohistochemistry, the large histiocytes showed strong positive staining for S100, CD163, and cyclin D1, while lacking significant expression of BRAF V600E, consistent with Rosai-Dorfman disease of the soft tissue/bone (Fig. 3B).

**Fig. 4:**
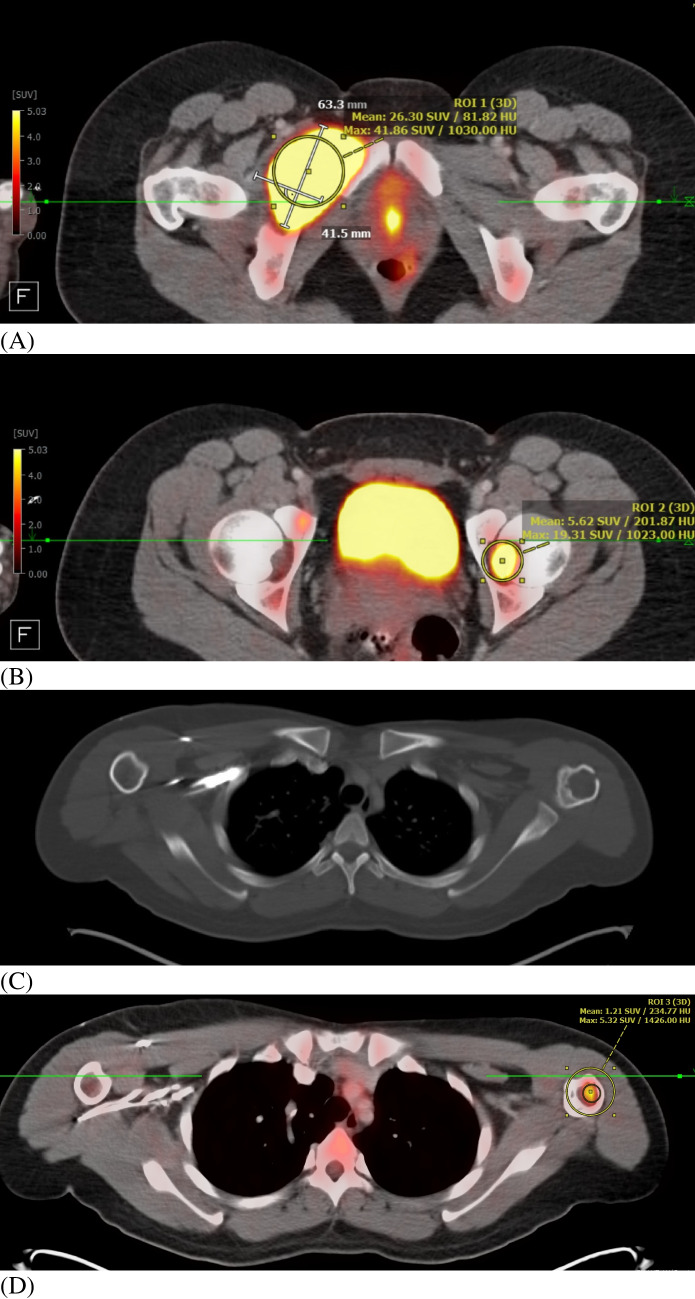
FDG PETCT with diagnostic CT. **A** Lytic and soft tissue lesion involving the right superior pubic ramus and anterior acetabulum with a large soft tissue component extending into the adductor muscles and extending into the anterior inferior aspect of the hip joint/acetabular fossa with intense FDG uptake, measuring up to 63 × 42 mm SUVmax of 41.9. **B** Soft tissue lesion centered in the left femoral head with cortical breakthrough and involvement of the left hip joint with intense FDG uptake SUVmax of 19.3. **C** Lucent lesion with focal intense FDG uptake and focal cortical breakthrough in the left proximal humerus (arrow). **D** Left proximal humerus lesion demonstrates focal intense FDG uptake SUVmax of 5.3

An FDG PET CT with diagnostic CT was performed to assess the radiographic extent of the disease (Fig. 4). A lytic lesion involving the right superior pubic ramus and anterior acetabulum with a large soft tissue component extending into the adductor muscles and extending into the anterior inferior aspect of the hip joint/acetabular fossa measuring up to 63 × 42 mm demonstrated intense FDG uptake. An additional soft tissue lesion was identified centered in the left femoral head with cortical breakthrough and involvement of the left hip joint with intense FDG uptake and SUVmax of 19.3 and corresponding to finding on prior MRI. A third lucent lesion with focal intense FDG uptake and focal cortical breakthrough in the left proximal humerus was also revealed (Fig. 4D). There was no lymphadenopathy by CT size criteria or lymph nodes with significant FDG uptake.

## Discussion

**Fig. 5:**
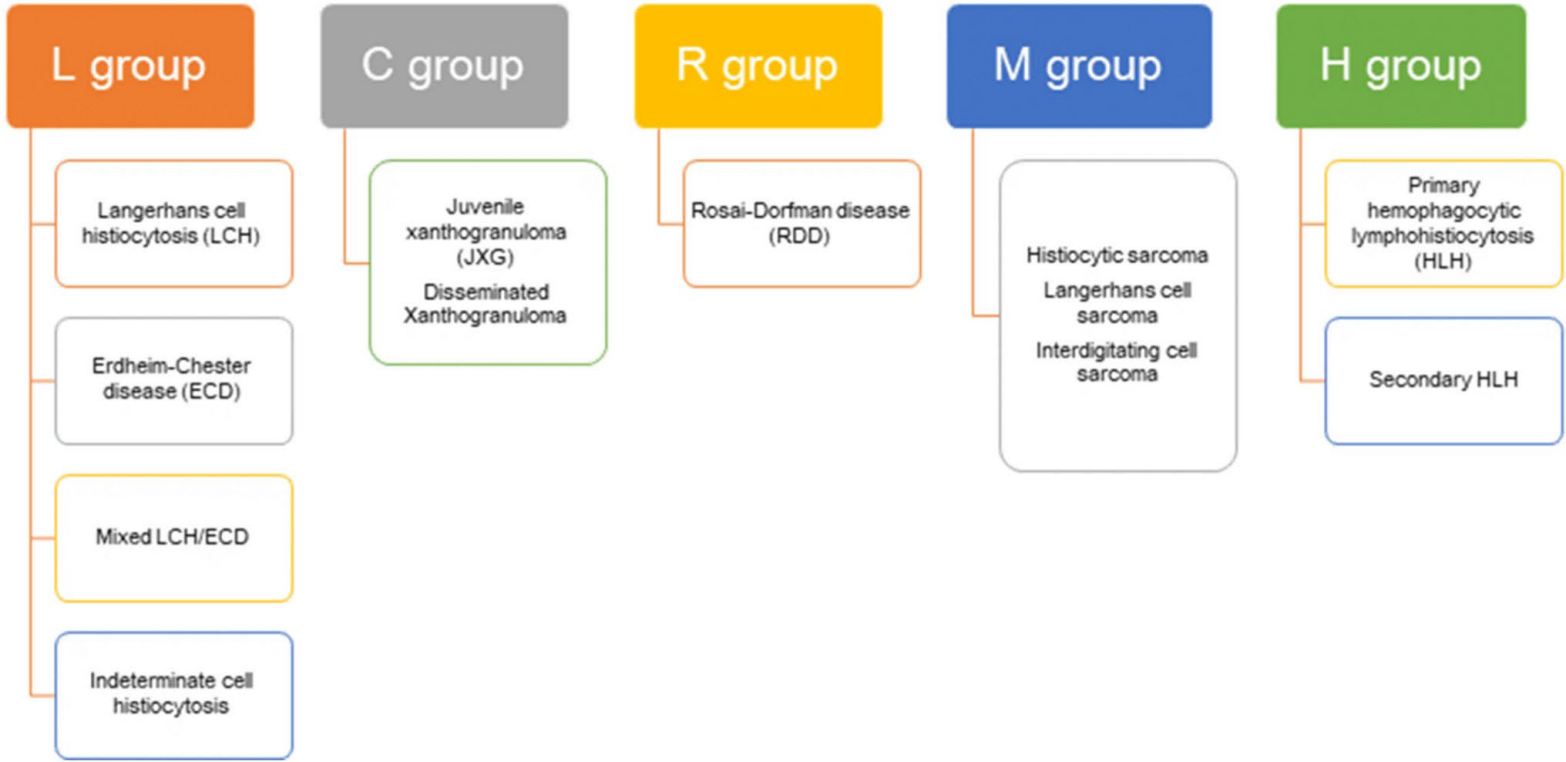
Revised classification of histiocytic disorders by the Histocyte Society [12] based on cell origins, molecular pathogenesis, and clinical presentations. There are five main groups based on cell origins, molecular pathogenesis, and clinical presentations, including Langerhans related (L group), cutaneous and mucocutaneous histiocytosis (C group), Rosai-Dorfman disease (R group), malignant histiocytosis (M group), and hemophagocytic lymphohistiocytosis and macrophage activation syndrome (H group)

Since its initial description in 1969 by Rosai and Dorfman, understanding of the pathophysiology of Rosai-Dorfman disease continues to evolve. RDD falls under the spectrum of histiocytic disorders which includes Langerhans histiocytosis and Erdheim-Chester disease. It is now classified under its own subtype (“R group”) under a recently revised classification of histiocytic disorders and neoplasms based on characteristics of macrophage-dendritic cell lineage [1]. This classification is based on a combination of cell origins, clinical presentations, and molecular pathogenesis (Fig. 5).

RDD is a rare disease that most frequently occurs in young adults with a mean age of presentation at 20 years and with a slight male predilection [2]. The typical clinical presentation involves massive painless cervical lymphadenopathy with constitutional symptoms. Additional symptoms vary depending on the extent of extranodal involvement which can occur up to 40% [2, 3]. Osseous involvement has been estimated to occur in up to 10% of cases [2, 4]. Primary osseous RDD is an even rarer entity as mentioned previously and is estimated to be seen in only 2–8% of cases [2, 4, 5]. Within the literature, primary osseous RDD typically is solitary but polyostotic cases have also been described [13–15].

Literature review of imaging characteristics of osseous RDD reveals non-specific features such as cortical thinning, focal cortical breakthrough with soft tissue component, internal septations, and rare periosteal reactions. These non-specific aggressive and non-aggressive features make diagnosis challenging. Maintaining a high index of suspicion and tissue sampling with histological and immunohistochemical analysis are often a core component of diagnosis. Fine needle aspiration and large core needle biopsies have been documented to diagnosis osseous RDD without lymphadenopathy [16, 17]. Because of the non-uniformly distributed histiocytes throughout the lesion needed for diagnosis, biopsy results may sometimes lead to an erroneous diagnosis of osteomyelitis. Clinical correlation and multidisciplinary collaboration between the clinician, radiologist, surgeon, and pathologist are essential to determine the appropriate further work-up to make the correct diagnosis.

RDD typically follows a benign and self-limiting course with death occurring in less than 3% of cases which is usually due to systemic infection from impaired immune response [18]. The mainstay of treatment for asymptomatic nodal disease is careful observation with spontaneous remission sometimes occurring [18, 19]. Symptomatic bone lesions may require block resection or curettage with grafting or prophylactic internal fixation. Second-line treatments including chemotherapy, long-term corticosteroid therapy, and radiation therapy have also been described [20, 21].

Per chart review, the patient continued to experience right and some occasional left hip pain but was still able to complete a 5 K walk/run. Due to concern for possible instability of the right hip, intervention was recommended. Treatment included localized radiotherapy and continued careful observation of the femoral and humeral lesions. Due to the patient’s young age and the unavoidable risk of pelvic radiation affecting the ovaries, ovarian cryopreservation was suggested before initiating local radiation therapy. Surgical excision and systemic treatment were also discussed. Options for systemic treatment include MEK inhibitors such as cobimetinib or immunosuppressive chemotherapy with steroids, rituximab, vincristine, or cladribine. Ultimately, the patient was subsequently lost to follow-up.

Polyostotic RDD without nodal involvement is extremely rare. Its nonspecific clinical presentation and imaging findings can resemble those of aggressive conditions, necessitating proper tissue sampling and correlation with imaging findings for an accurate diagnosis. Considering the patient’s age and demographics is crucial for maintaining a broad differential diagnosis, facilitating correct identification and appropriate management, which may include additional measures such as ovarian cryopreservation, as exemplified in our case.

## Data Availability

Not applicable.

## References

[1] Emile JF, Abla O, Fraitag S, Horne A, Haroche J, Donadieu J, et al. Revised classification of histiocytoses and neoplasms of the macrophage-dendritic cell lineages. Blood. 2016;127(22):2672–81. 26966089 10.1182/blood-2016-01-690636 PMC5161007

[2] Foucar E, Rosai J, Dorfman R. Sinus histiocytosis with massive lymphadenopathy (Rosai-Dorfman disease): review of the entity. Semin Diagn Pathol. 1990;7(1):19–73. 2180012

[3] McAlister WH, Herman T, Dehner LP. Sinus histiocytosis with massive lymphadenopathy (Rosai-Dorfman disease). Pediatr Radiol. 1990;20(6):425–32. 2202971 10.1007/BF02075199

[4] Duijsens HM, Vanhoenacker FM, ter Braak BP, Hogendoorn PC, Kroon HM. Primary intraosseous manifestation of Rosai-Dorfman disease: 2 cases and review of literature. JBR-BTR. 2014;97(2):84–9. 25073237 10.5334/jbr-btr.18

[5] Demicco EG, Rosenberg AE, Björnsson J, Rybak LD, Unni KK, Nielsen GP. Primary rosai-dorfman disease of bone: a clinicopathologic study of 15 cases. Am J Surg Pathol. 2010;34(9):1324–33. 20679880 10.1097/PAS.0b013e3181ea50b2

[6] Choraria A, Andrei V, Rajakulasingam R, Saifuddin A. Musculoskeletal imaging features of non-Langerhans cell histiocytoses. Skeletal Radiol. 2021;50(10):1921–40. 33787962 10.1007/s00256-021-03765-0

[7] Ross AB, Davis KW, Buehler D, Chan BY. Primary Rosai-Dorfman disease of bone: a report of two cases. Case Rep Radiol. 2019;2019:1720131. 30719368 10.1155/2019/1720131 PMC6335665

[8] George J, Stacy G, Peabody T, Montag A. Rosai-Dorfman disease manifesting as a solitary lesion of the radius in a 41-year-old woman. Skeletal Radiol. 2003;32(4):236–9. 12652340 10.1007/s00256-002-0613-x

[9] Safavi M, Panjavi B, Pak N. Rosai-Dorfman disease arising in patella. Arch Iran Med. 2019;22(12):731–2. 31823626

[10] Patterson FR, Rooney MT, Damron TA, Vermont AI, Hutchison RE. Sclerotic lesion of the tibia without involvement of lymph nodes. Report of an unusual case of Rosai-Dorfman disease. J Bone Jt Surg Am. 1997;79(6):911–6. 10.2106/00004623-199706000-00017 9199391

[11] Abdelwahab IF, Klein MJ, Springfield DS, Hermann G. A solitary lesion of talus with mixed sclerotic and lytic changes: Rosai-Dorfman disease of 25 years’ duration. Skeletal Radiol. 2004;33(4):230–3. 14740182 10.1007/s00256-003-0681-6

[12] Histiocytosis Association. Overview of histiocytic disorders [Internet]. 2021 [cited 2024 Nov 4]. Available from: https://histio.org/histiocytic-disorders/

[13] Grote HJ, Moesenthin M, Foss HD, Kekow J, Roessner A. Osseous manifestation of Rosai-Dorfman disease (sinus histiocytosis with massive lymphadenopathy). A case report and review of the literature. Gen Diagnostic Pathol. 1998;143(5–6):341–5. 9653919

[14] Park YK, Kim YW, Choi WS, Lim YJ. Sinus histiocytosis with massive lymphadenopathy - Multiple skull involvements. J Korean Med Sci. 1998;13(4):423–7. 9741548 10.3346/jkms.1998.13.4.423 PMC3054428

[15] Goel MM, Agarwal PK, Agarwal S. Primary Rosai-Dorfman disease of bone without lymphadenopathy diagnosed by fine needle aspiration cytology: a case report. Acta Cytol. 2003;47(6):1119–22. 14674094 10.1159/000326661

[16] Ruggiero A, Attinà G, Maurizi P, Mulè A, Tarquini E, Barone G, et al. Rosai-Dorfman disease: two case reports and diagnostic role of fine-needle aspiration cytology. J Pediatr Hematol Oncol. 2006;28(2):103–6. 16462585 10.1097/01.mph.0000200686.33291.d1

[17] Pulsoni A, Anghel G, Falcucci P, Matera R, Pescarmona E, Ribersani M, Villivà N, Mandelli F. Treatment of sinus histiocytosis with massive lymphadenopathy (Rosai-Dorfman disease): report of a case and literature review. Am J Hematol. 2002;69(1):67–71. 10.1002/ajh.10008. 11835335 10.1002/ajh.10008

[18] Lin J, Lazarus M, Wilbur A. Sinus histiocytosis with massive lymphadenopathy: MRI findings of osseous lesions. Skeletal Radiol. 1996;25:279–82. 8741068 10.1007/s002560050080

[19] Paryani NN, Daugherty LC, O’Connor MI, Jiang L. Extranodal Rosai-Dorfman disease of the bone treated with surgery and radiotherapy. Rare Tumors. 2014;6(4):132–4. 10.4081/rt.2014.5531 PMC4274442 25568748

[20] Saboo SS, Jagannathan JP, Krajewski KM, O’Regan K, Hornick JL, Fisher DC, et al. Symptomatic extranodal Rosai-Dorfman disease treated with steroids, radiation, and surgery. J Clin Oncol. 2011. 10.1200/JCO.2011.36.9967. 10.1200/JCO.2011.36.9967 21931038

[21] Sundaram C, Shantveer GU, Chandrashekar P, Prasad VBN, Umadevi M. Multifocal osseous involvement as the sole manifestation of Rosai-Dorfman disease. Skeletal Radiol. 2005;34:658–64. 16094546 10.1007/s00256-005-0951-6

